# Stable QTL Identification and KASP Marker Development for Grain Protein Content in Wheat Using a Linmai2/Zhong892 RIL Population

**DOI:** 10.3390/plants15152307

**Published:** 2026-07-27

**Authors:** Nannan Zhao, Yamei Wang, Yuxuan Song, Ruitong Shi, Ye Lian, Qiang Li, Chunyan Xie

**Affiliations:** 1 College of Life Sciences, Langfang Normal University, Langfang 065000, China; hbndnannan@163.com (N.Z.); wangyamei23@126.com (Y.W.); 18531015925@163.com (Y.S.); 13561484020@163.com (R.S.); 15333097226@163.com (Y.L.); 2 Dryland Farming Institute, Hebei Academy of Agriculture and Forestry Sciences, Hengshui 053000, China

**Keywords:** common wheat, GPC, quality, KASP, SNP

## Abstract

Grain protein content (GPC) is extremely important for common wheat (*Triticum aestivum* L.) end-use quality. Identifying genetic loci for GPC is helpful in creating new varieties with good processing quality. Linmai2 and Zhong892 are two elite wheat varieties with differences in GPC. In the present study, a recombinant inbred line (RIL) population derived from Linmai2 and Zhong892 was used to identify GPC loci. Linkage mapping was conducted by combining the wheat 90K single nucleotide polymorphisms (SNPs) chip data and GPC from four environments. Five QTLs on chromosomes 1B, 2B, 4A, 4B and 6B were identified, and designated as *QGPC.lfnu-1BL*, *QGPC.lfnu-2BS*, *QGPC.lfnu-4AS*, *QGPC.lfnu-4B* and *QGPC.lfnu-6BS*, respectively, explaining 5.21–11.38% of the phenotypic variances. The favorable alleles of *QGPC.lfnu-1BL*, *QGPC.lfnu-4AS*, *QGPC.lfnu-4B* and *QGPC.lfnu-6BS* originated from Zhong892, while the favorable allele of *QGPC.lfnu-2BS* came from Linmai2. Among these, *QGPC.lfnu-1BL*, *QGPC.lfnu-2BS*, *QGPC.lfnu-4AS*, and *QGPC.lfnu-4B* are potentially novel loci, whereas *QGPC.lfnu-6BS* likely represents the re-detection of *Gpc-B1*. Additionally, competitive allele-specific PCR (KASP) markers suitable for breeding were developed, and genetic effects were validated in a natural population. Four KASP markers, *KASP-GPC-1BL*, *KASP-GPC-2BS*, *KASP-GPC-4B* and *KASP-GPC-6BS*, were significantly associated with GPC in a diverse panel of 140 wheat varieties. A total of twelve potential genes involved in nitrogen assimilation, amino acid biosynthesis and transport, and NPF transporter and protein turnover were proposed as candidate genes within the intervals of five QTLs. These findings identified stable QTLs, available KASP markers and promising candidate genes for marker assisted selection to accelerate wheat GPC improvement.

## 1. Introduction

Common wheat (*Triticum aestivum* L.) is one of the most widely cultivated staple crops globally, covering diverse agroecological zones from temperate to subtropical regions. As the primary food source for approximately 35–40% of the world’s population, wheat provides about 20% of daily caloric intake and 20–25% of dietary protein, underscoring its critical role in global food and nutritional security [1,2]. China is the world’s largest wheat producer and consumer, and the sustainable development of wheat production is of great significance for ensuring national food security [3]. Rising living standards and evolving consumer preferences have driven a substantial increase in the demand for high-quality wheat with superior processing attributes. However, compared with developed countries and market requirements, China’s production of high-quality wheat still faces considerable challenges, including a limited number of elite varieties, inconsistent quality stability, and heavy dependence on imports [4]. Consequently, grain quality improvement has become an urgent priority in Chinese wheat breeding programs [5].

Wheat grain protein content (GPC) is a key parameter for evaluating the intrinsic quality and processing performance of wheat, directly affecting the end-use quality and market value of flour [6]. GPC serves as a core indicator for wheat quality grading and exerts a profound influence on dough rheological properties, including mixing tolerance, viscoelasticity, and gluten strength [7]. Higher GPC generally correlates with stronger gluten, which is desirable for bread-making, whereas moderate protein content is preferred for noodle and pastry production. Variations in GPC influence water absorption, dough stability, and fermentation properties, thereby affecting the texture, volume, and consumer acceptability of end-use products such as bread, noodles, and steamed buns [7]. Elucidating the genetic basis of GPC is essential for improving wheat quality, enabling breeders to develop new varieties that combine high yield and excellent processing quality to meet the specific demands of different regions and markets [8].

GPC is a typical quantitative trait controlled by multiple minor-effect genes and is significantly influenced by environmental factors [9]. Previous quantitative trait locus (QTL) mapping studies have reported that GPC-associated QTLs are distributed on all wheat chromosomes, with major loci frequently identified on 2A, 2B, 2D, 3D, 5A, 6A, 6B, and 7B [10,11]. One of the most notable examples is *Gpc-B1* on chromosome 6BS, a major QTL that increases GPC without yield penalty [12]. Other stable QTLs have been detected in diverse genetic backgrounds, such as *QGPC.ndsu.1A.1*, explaining 16.5% of phenotypic variance, and *QLGPC.cau-1B*, which was identified under multiple growing conditions [13,14,15]. However, most of these studies relied on low-density simple sequence repeat (SSR) or diversity array technology (DArT) markers, resulting in broad QTL intervals and limited utility for gene cloning and marker-assisted selection (MAS) [16].

Beyond traditional biparental QTL mapping, genome-wide association studies (GWAS) [17,18], meta-QTL integration and genomic prediction have become mainstream tools for dissecting GPC genetic architecture in recent years [19]. Large-scale GWAS panels capture natural allelic variation to detect minor-effect loci overlooked in RIL populations, while meta-QTL analysis aggregates hundreds of independent mapping datasets to refine consensus stable GPC intervals and narrow candidate gene regions. Genomic prediction further enables accurate pre-selection of grain protein performance at early breeding generations without costly phenotypic assays. These multi-omics strategies complement biparental linkage mapping and accelerate the dissection of complex quality traits such as GPC. Competitive allele-specific PCR (KASP) is a cost-effective, high-throughput genotyping technology that enables precise discrimination of single nucleotide polymorphism (SNP) alleles. KASP markers have been widely adopted for QTL validation, fine mapping, and MAS in wheat breeding due to their flexibility, low cost per sample, and scalability [20,21,22]. The development of high-density SNP arrays (e.g., wheat 55 K, 90 K, and 660 K) has further facilitated the identification of reliable QTLs and the conversion of significant SNPs into functional KASP markers for practical breeding applications [20,23,24]. Multiple breeding-oriented studies have validated the efficiency of KASP markers for wheat quality improvement: diagnostic KASP assays targeting gluten strength, grain hardness and *Gpc-B1* have been widely deployed in Australian, Chinese and European wheat breeding pipelines to pyramid favorable quality alleles and eliminate inferior haplotypes in early segregating generations [25,26].

In this study, we utilized a recombinant inbred line (RIL) population comprising 262 lines derived from a cross between Zhong892, a germplasm with high GPC, and Linmai2, a variety with conventional processing quality. The RIL population provides an ideal mapping platform for dissecting the genetic architecture of GPC. Using a high-density genetic map constructed from genome-wide SNP markers, we performed comprehensive linkage analysis to identify QTLs associated with GPC. Our specific objectives were: (1) to detect stable QTLs controlling GPC in the Linmai2/Zhong892 RIL population; (2) to predict candidate genes within the mapped intervals through comparative genomic analysis; and (3) to develop KASP markers for MAS in wheat quality breeding. The findings from this study are expected to provide new genetic resources and molecular tools for accelerating the improvement of wheat grain protein quality.

## 2. Results

### 2.1. Phenotypic Evaluation

**Figure 1:**
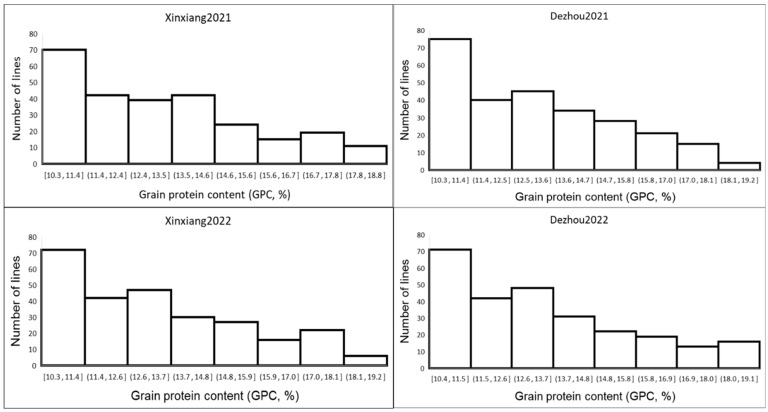
The distribution of the grain protein content in Linmai2/Zhong892 RIL population. E1: Xinxiang2021, E2: Xinxiang2022, E3: Dezhou2021 and E4 Dezhou2022.

The GPC of the Zhong892 (high GPC) × Linmai2 (low GPC) RIL population comprising 262 lines was evaluated across four environments. The parental lines exhibited distinct GPC levels, and the RIL population displayed wide and continuous variation. Across all environments, GPC ranged from 10.30% to 19.23%. The mean GPC values were 13.28% (E1, Xinxiang2021), 13.30% (E2, Xinxiang2022), 13.22% (E3, Dezhou2021), and 13.33% (E4, Dezhou2022), with standard deviations of 2.30%, 2.35%, 2.22%, and 2.38%, respectively (Table 1; Figure 1). Coefficients of variation ranged from 17.0% to 18.0%, indicating a moderate degree of phenotypic variability. Transgressive segregation was pronounced, with many lines surpassing both the low and high parental values. The frequency distributions of GPC were approximately normal and continuous in all environments, which is consistent with polygenic inheritance and suitable for QTL analysis. The consistent phenotypic distribution across environments indicated moderate genetic control of GPC, consistent with the moderate broad-sense heritability (*H*_b_^2^ = 0.68). These phenotypic data provided a solid foundation for subsequent QTL mapping. Pearson’s correlation coefficients for GPC across the four environments ranged from 0.66 to 0.88 (*p* < 0.05), indicating strong positive correlations. Analysis of variance (ANOVA) revealed that genotype, environment, and their interaction all had significant effects on GPC, with genotype accounting for the largest proportion of variance (Table 2). Broad sense heritability (*H*_b_^2^) for GPC was estimated to be 0.63 across environments.

### 2.2. Linkage Mapping of Grain Protein Content

**Figure 2:**
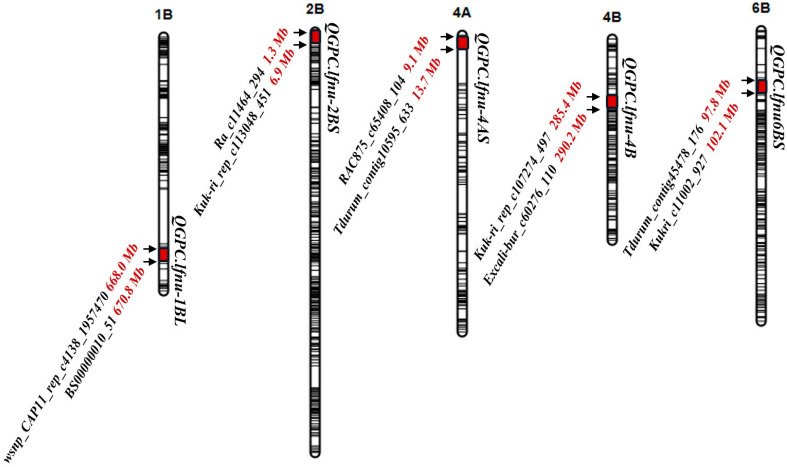
The QTL identified in the Linmai2/Zhong892 RIL population.

QTL mapping for GPC was performed using a Zhong892 × Linmai2 RIL population across four environments. Five QTLs were identified on chromosomes 1BL, 2BS, 4AS, 4B, and 6BS, with LOD scores of 2.89–8.82, explaining 5.21–11.38% of phenotypic variance. They were designated *QGPC.lfnu-1BL*, *QGPC.lfnu-2BS*, *QGPC.lfnu-4AS*, *QGPC.lfnu-4B*, and *QGPC.lfnu-6BS* (Table 3; Figure 2).

*QGPC.lfnu-1BL* was identified on chromosome 1BL in environments Xinxiang21 and Xinxiang2022, flanked by markers *wsnp_CAP11_rep_c4138_1957470* and *BS00000010_51* (668.0–670.8 Mb). It had a LOD score of 3.68 and explained 6.12–6.65% of the GPC variation, with an additive effect of 0.6–0.7. The favorable allele, which increased GPC, was inherited from Zhong892. *QGPC.lfnu-2BS* mapped to chromosome 2BS, was expressed in three environments (Xinxiang2021, Xinxiang2022, Dezhou2021 and BLUE), lying between markers *Ra_c11464_294* and *Kukri_rep_c113048_451* (1.3–6.9 Mb). This QTL showed a LOD of 2.89 and a PVE of 5.62–5.99%, with an additive effect of −0.5–0.6. Interestingly, the favorable allele originated from the low-GPC parent Linmai2, indicating that Zhong892 contributed a GPC-reducing allele at this locus. On chromosome 4AS, *QGPC.lfnu-4AS* was detected in Xinxiang2022 and Dezhou2021, with a genetic interval defined by *RAC875_c65408_104* and *Tdurum_contig10595_633* (9.1–13.7 Mb). It had a LOD of 3.05, explained 5.21–5.78% of the PVE, and showed an additive effect of 0.7–0.8, with the favorable allele coming from Zhong892. The most stable, relatively large-effect QTL was *QGPC.lfnu-4B* on chromosome 4B, detected in all four environments and BLUE. It was located between *Kukri_rep_c107274_497* and *Excalibur_c60276_110* (285.4–290.2 Mb). This QTL exhibited the highest LOD score of 8.82 and explained the largest proportion of PVE (7.63–11.38%), with an additive effect of 1.0–1.12. The favorable allele was contributed by Zhong892, making *QGPC.lfnu-4B* a promising candidate for fine mapping and marker-assisted selection. Finally, *QGPC.lfnu-6BS* was mapped to chromosome 6BS in environments Xinxiang2022 and Dezhou2022 and was flanked by markers *Tdurum_contig45478_176* and *Kukri_c11002_927* (97.8–102.1 Mb). It had a LOD of 4.26, explained 6.21–7.25% of the PVE, and showed an additive effect of 0.7–0.8, with the favorable allele derived from Zhong892. In summary, four of the five QTLs (1BL, 4AS, 4B, and 6BS) carried GPC-increasing alleles from the high-GPC parent Zhong892, whereas only *QGPC.lfnu-2BS* received its protein-enhancing allele from the low-GPC parent Linmai2. The physical intervals of these QTLs ranged from approximately 2.8 Mb to 5.6 Mb. These stable QTLs, particularly *QGPC.lfnu-4B*, represent valuable targets for improving GPC in wheat breeding programs.

### 2.3. Prediction of Candidate Genes for GPC

**Figure 3:**
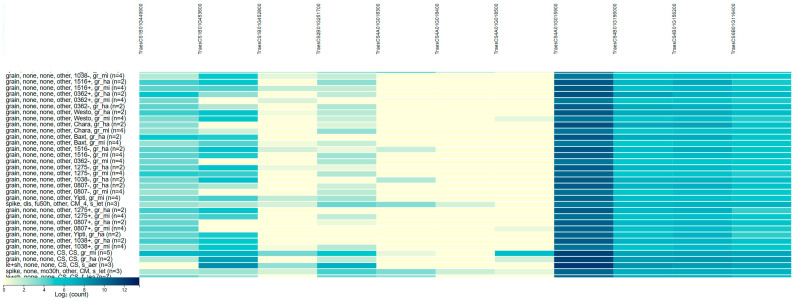
The expression pattern for the candidate gene by the expVIP database (www.wheat-expression.com/ accessed on 22 July 2026).

A total of 273 high-confidence genes were annotated across the five QTL physical intervals (Table A2). Functional annotation of the physical intervals corresponded to five stable QTLs and identified twelve candidate genes putatively involved in GPC. These genes cover key processes such as amino acid biosynthesis, nitrogen transport, protein turnover, and carbon-nitrogen balance. Within *QGPC.lfnu-1BL*, three genes were predicted: *TraesCS1B01G449900*, *TraesCS1B01G453600* and *TraesCS1B01G452800*. The *QGPC.lfnu-2BS* interval contained one candidate, *TraesCS2B01G251700*, encoding an NPF transporter. The *QGPC.lfnu-4AS* interval contained five candidates: *TraesCS4A01G016900*, *TraesCS4A01G018300*, *TraesCS4A01G018400*, *TraesCS4A01G018500* and *TraesCS4A01G015900*. In addition, two candidate genes (*TraesCS4B01G156000*, encoding E3 ubiquitin protein ligase, and *TraesCS4B01G156200*, encoding a PPR protein) for *QGPC.lfnu-4B* were identified. For *QGPC.lfnu-6BS*, a single candidate, *TraesCS6B01G116400*, was identified (Table 4; Figure 3).

### 2.4. KASP Marker Development and Validation

**Figure 4:**
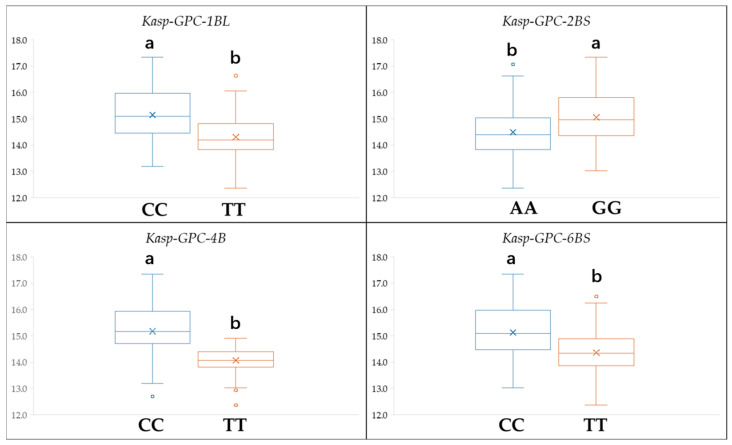
Box-plot of the KASP marker in the 140 wheat accessions. Different letters between boxes indicate significance at *p* < 0.05.

To validate QTL effects and enable MAS breeding, KASP markers for four major GPC QTLs were developed and genotyped across 140 wheat varieties (Table 5 and Table A3). Allelic groups were compared by t-test, and all four markers showed significant associations with GPC (*p* < 0.05). For *QGPC.lfnu-1BL*, *KASP-GPC-1BL* distinguished CC and TT alleles: 74 CC-carrying varieties had a mean GPC of 15.0%, 0.5 percentage points higher than 66 TT varieties (14.5%), which is consistent with the favorable CC allele derived from the high-GPC parent Zhong892. At *QGPC.lfnu-2BS*, *KASP-GPC-2BS* identified AA and GG alleles. The AA allele (65 varieties) conferred higher GPC (15.0%) than the GG allele (14.4%, 75 varieties), indicating that AA is the protein-elevating allele at this locus. For the stable major QTL *QGPC.lfnu-4B*, *KASP-GPC-4B* revealed TT and CC alleles. The CC allele (61 varieties) was associated with 15.0% GPC, significantly exceeding the 14.5% of TT (79 varieties), a 0.5% effect mirroring the RIL population and supporting CC as the Zhong892-derived superior variant. At *QGPC.lfnu-6BS*, *KASP-GPC-6BS* showed TT/CC polymorphism: the CC group (70 varieties) averaged 15.1% GPC versus 14.4% for TT (70 varieties), a 0.7% difference, agreeing with Zhong892 providing the high-GPC allele (Table 6). In summary, the KASP markers clearly discriminated high- and low-protein alleles across the panel. The absolute GPC differences between alleles ranged from 0.5% to 0.7%, representing substantial breeding value. The complete correspondence between the KASP results and the original QTL findings demonstrates the robustness of these loci and confirms that these markers exhibit reliable phenotypic association in the tested diversity panel and show promising potential for MAS; further independent validation in segregating breeding populations is required to confirm their consistent genetic effects across diverse genetic backgrounds before large-scale breeding application. (Table 6, Figure 4).

The 140 wheat varieties used for KASP validation are all pure-line cultivars derived from repeated self-pollination and are thus expected to be highly homozygous. Furthermore, in such a strictly self-pollinated crop, residual heterozygosity in elite varieties is extremely rare; with a validation panel of 140 accessions, the statistical power to detect such low-frequency heterozygous events is inherently limited. In addition, KASP genotype calling employs conservative clustering algorithms that assign borderline signals to homozygous clusters to ensure data quality. Consistently, our KASP genotyping results showed only homozygous allelic calls for each marker across the entire panel.

## 3. Discussion

Enhancing grain quality remains a central objective in wheat improvement programs. While considerable effort is devoted to boosting yield potential, equal emphasis is placed on end-use traits, among which GPC is a key determinant of processing quality and nutritional value. Although environmental factors, agronomic practices, and soil fertility exert substantial influence on this trait, the broad phenotypic diversity observed among wheat lines points to a significant genetic component. With the rapid development of genomics and molecular breeding tools [27,28,29,30], the dissection of complex quality traits such as GPC has become increasingly tractable. The integration of QTL detection, meta-analyses, and transcriptome profiling now enables the systematic identification of robust loci and underlying candidate genes. Harnessing this natural variation through molecular strategies offers an effective pathway for quality improvement that is compatible with sustainable agriculture. We performed linkage analysis for GPC in a Linmai2/Zhong892 RIL population. Five genomic regions controlling GPC were mapped. Notably, *QGPC.lfnu-4B* stood out as the locus consistently expressed across all four environments, accounting for the largest proportion of phenotypic variation (11.38%). We developed validated KASP markers to support marker-assisted selection aimed at elevating grain protein levels.

### 3.1. Comparisons with Previous Reports

GPC QTLs have been reported previously on all wheat chromosomes. Genetic dissection of GPC has been a long-standing focus in wheat research, and numerous QTLs have been documented across all 21 chromosomes [31,32,33]. In our study, five QTLs were identified on chromosomes 1BL, 2BS, 4AS, 4B, and 6BS. To assess the novelty of these loci, we compared their chromosomal positions and physical intervals with previously reported GPC QTLs (Table A1).

*QGPC.lfnu-6BS* (97.8–102.1 Mb on 6BS) overlaps the physical position of the cloned *NAM-B1* (*Gpc-B1*) transcription factor (99.1–99.5 Mb, *Xuhw89* and *Xgwm219*), which is well documented to accelerate leaf senescence and nitrogen remobilization to grains. The consistent physical overlap confirms that this detected locus corresponds to the canonical *Gpc-B1* locus originating from wild emmer wheat [12,34]. *Gpc-B1*, a NAC transcription factor (*NAM-B1*), accelerates leaf senescence and promotes nitrogen remobilization into developing grains, leading to higher GPC [12,34]. The physical position and functional mechanism of our QTL coincide with *Gpc-B1*, suggesting that the favorable allele from Zhong892 might be an allelic variant of *NAM-B1* or a tightly linked gene with a similar effect. The identification of this locus in a bread wheat background confirms its value and provides a direct target for marker-assisted selection without the yield penalty often associated with *Gpc-B1* in certain environments [34]. For *QGPC.lfnu-2BS*, although GPC QTLs on chromosome 2B have been reported in several studies [31,33], most were mapped to the long arm or in different regions of the short arm. The physical interval of 1.3–6.9 Mb on 2BS is relatively distal, and to our knowledge no major GPC locus has been precisely localized to this segment. Moreover, the favorable allele was contributed by the low-GPC parent Linmai2, indicating transgressive segregation and suggesting a unique allele that increases GPC without coming from a high-protein donor. *QGPC.lfnu-2BS* is a potentially new and useful locus for GPC improvement (Table A1).

*QGPC.lfnu-1BL*, mapped to the terminal 668.0–670.8 Mb region of chromosome 1BL, also appears to be novel. While 1BL harbors several reported GPC QTLs, the majority cluster in more proximal bins [31,35], and the physical interval defined here is distinct. Thus, *QGPC.lfnu-1BL* likely represents a new GPC locus, expanding the genetic resources available for the long arm of chromosome 1B. *QGPC.lfnu-4AS* lies within a 4.6-Mb physical interval on the short arm of chromosome 4A (9.1–13.7 Mb) (Table A1). A few GPC QTLs have been reported on 4A, but they mainly reside on the long arm or proximal parts of the short arm [32,35]. Therefore, our QTL occupies a region that has not been frequently associated with GPC, making it a strong candidate for a novel locus. The presence of multiple amino acid transporter-like genes in this interval strengthens the hypothesis that it plays a direct role in amino acid loading into the grain.

Finally, the most prominent QTL, *QGPC.lfnu-4B*, was consistently detected across all four environments and explained over 11% of phenotypic variance. The physical interval (285.4–290.2 Mb) maps to the central region of chromosome 4B. Although a few reports have indicated GPC QTLs on 4B [31], very few have been validated in multiple environments and none in this specific interval with such a large effect. Given its stability, the magnitude of its additive effect (1.12%), and the absence of comparable references, we propose *QGPC.lfnu-4B* as a novel major GPC locus that holds exceptional promise for wheat quality breeding. In conclusion, *QGPC.lfnu-1BL*, *QGPC.lfnu-2BS, QGPC.lfnu-4AS*, and *QGPC.lfnu-4B* are potential novel loci, whereas *QGPC.lfnu-6BS* likely represents the re-detection of *Gpc-B1* in our germplasm. The novel QTLs substantially enrich the genetic pool for GPC improvement [12,34,35]. These findings highlight the value of diverse elite cultivars in uncovering new genetic variation and provide solid targets for further fine-mapping and gene cloning.

### 3.2. Potential Candidate Genes

In this study, a total of twelve candidate genes putatively involved in wheat GPC were identified within five stable QTL intervals. These genes are implicated in diverse yet interconnected biological processes, including amino acid biosynthesis, nitrogen transport and remobilization, transmembrane transport, protein turnover, and carbon–nitrogen balance, thereby providing valuable molecular targets for dissecting the genetic architecture of GPC. Within the *QGPC.lfnu-1BL* interval, three candidate genes were predicted. *TraesCS1B01G449900* encodes 4-hydroxy-tetrahydrodipicolinate synthase (DHDPS), a rate-limiting enzyme in lysine biosynthesis [36]. By modulating the free amino acid pool available for storage protein synthesis, DHDPS may directly influence GPC. *TraesCS1B01G453600* encodes asparagine synthetase, which catalyzes the formation of asparagine, a major nitrogen transport and storage compound, thereby facilitating nitrogen remobilization from source tissues to developing grains [37]. *TraesCS1B01G452800* encodes a carboxypeptidase involved in protein degradation and turnover [38]. Although proteolysis is often associated with net protein loss, regulated turnover during late grain development may modulate the final protein content, making this gene a potentially important modulator rather than a simple negative regulator. *TraesCS2B01G251700* for *QGPC.lfnu-2BS* encodes an NPF transporter, which may facilitate the transmembrane movement of amino acids into developing grains, which is consistent with the role of NPF members in nitrogen remobilization during grain filling [39]. The *QGPC.lfnu-4AS* contained five candidate genes. Among them, *TraesCS4A01G016900* encodes 3-dehydroquinate synthase (DHQS), which catalyzes the first committed step of the shikimate pathway. This pathway provides precursors for the biosynthesis of aromatic amino acids (phenylalanine, tyrosine and tryptophan). Notably, three contiguous genes, *TraesCS4A01G018300*, *TraesCS4A01G018400* and *TraesCS4A01G018500*, all encode amino acid transporter-like proteins [34,35,40,41,42]. Their tandem arrangement suggests cooperative action in mediating the transmembrane transport of amino acids into the endosperm, a critical step for protein deposition. Indeed, expression levels of such transporters are often positively correlated with the rate of grain nitrogen accumulation. Additionally, *TraesCS4A01G015900* encodes an aminotransferase-like protein that catalyzes the interconversion of nitrogenous intermediates, thereby influencing the abundance of glutamate-family amino acids and ultimately protein composition [43,44]. *TraesCS4B01G156000* for *QGPC.lfnu-4B* encodes an E3 ubiquitin ligase, implicating the ubiquitin–proteasome system in protein turnover and nitrogen-intermediate conversion, as previously shown for the wheat E3 ligase TaGW2 that affects both grain weight and protein content [45]. The adjacent *TraesCS4B01G156200* encodes a PPR/GR-RBP protein, which may influence organellar RNA metabolism and thus impact carbon/nitrogen assimilation, a role supported by the diverse functions of PPR proteins in plant development [46]. For *QGPC.lfnu-6BS*, *TraesCS6B01G116400* encodes β-amylase. As a starch-degrading enzyme, β-amylase may alter the carbon–nitrogen balance and source–sink relationships during grain filling. By modulating the supply of starch degradation products (e.g., maltose), it could indirectly affect nitrogen assimilation efficiency and the allocation of nitrogen to grain proteins [47,48]. This gene is particularly noteworthy because its genomic region overlaps with the previously fine-mapped *Gpc-B1* locus, a well-known regulator of GPC that operates through carbon–nitrogen metabolic coordination. Nevertheless, the inherent challenges in translating QTL intervals to definitive causal genes in common wheat, given its ~16-Gb genome, which makes direct candidate gene cloning from initial mapping extremely difficult. As the reviewer rightly points out, rigorous validation typically requires near-isogenic line (NIL)-based fine mapping, coupled with transcriptome profiling, followed by haplotype analysis across diverse germplasm to confirm phenotypic associations, and ultimately functional validation via transgenic complementation or CRISPR/Cas9-mediated gene editing. Only through such a stepwise pipeline can a candidate be conclusively proven. In the present study, our intention is not to claim definitive causal genes, but rather to provide a preliminary, evidence-based prediction that leverages gene annotation, public expression databases, and available orthologous knowledge from other cereals. This serves as a starting point for the community and offers testable hypotheses for future fine-mapping and functional studies.

### 3.3. Applications in Wheat Grain Quality Breeding

In wheat breeding, simultaneously improving grain yield and GPC is notoriously difficult because of their strong negative correlation. Traditional selection for quality traits relies heavily on labor intensive and time-consuming biochemical assays, while phenotypic selection for GPC alone often sacrifices yield. Recent advances in molecular genetics and genomics assisted breeding have provided efficient alternatives, and KASP markers, in particular, have become a robust, cost effective, and high throughput platform for MAS that operates independently of environmental conditions. In this study, four KASP markers (*KASP-GPC-1BL*, *KASP GPC 2BS*, *KASP GPC 4B*, and *KASP GPC 6BS*) were developed and validated in a panel of 140 wheat varieties, confirming that each marker clearly distinguished high and low GPC alleles with significant differences. The practical application of these markers allows breeders to efficiently pyramid multiple favorable alleles; based on the additive nature of these QTLs, combining all four elite alleles is expected to further elevate GPC without major compromise in yield.

ANOVA revealed that GPC is significantly influenced by both environment and G×E interaction, confirming its nature as an environment-sensitive quantitative trait. Among the five QTLs identified, the major locus *QGPC.lfnu-4B* was consistently detected across all four environments with the largest genetic effect and minimal environmental sensitivity, giving it the highest breeding application value. The remaining four QTLs showed only moderate stability (detected in 2–3 environments) and are more suitable as auxiliary selection targets; pyramiding these favorable alleles requires multi-environment screening to eliminate lines exhibiting strong negative G×E interaction. Given the moderate broad-sense heritability (0.68), phenotypic selection alone is inefficient. The KASP markers developed in this study can largely circumvent environmental interference and substantially improve selection accuracy for high-GPC lines—this represents the core advantage of the molecular tools established here.

It should be noted, however, that genotype by environment interactions and genetic background effects may influence the performance of these QTLs when transferred into new elite germplasm, so preliminary field validation in target environments is recommended. Furthermore, several RILs from the Zhong892 × Linmai2 population and superior varieties from the natural panel that carry favorable allele combinations at these loci also exhibited excellent agronomic performance and high GPC. These lines and cultivars represent valuable parental materials for quality focused wheat breeding programs, facilitating the simultaneous improvement of grain yield and protein content through molecular breeding strategies.

### 3.4. Future Perspectives

While our study has identified five stable QTLs and 12 candidate genes putatively associated with GPC, functional validation of these genetic determinants remains a critical next step. The candidate genes, particularly those involved in amino acid biosynthesis (*TraesCS1B01G449900*, *TraesCS4A01G016900*), nitrogen transport and remobilization (*TraesCS1B01G453600*, *TraesCS4A01G018300/4/5*), and carbon–nitrogen balance (*TraesCS6B01G116400*), represent promising targets for in-depth functional characterization. Future work should prioritize systematic validation using CRISPR/Cas9-mediated gene editing to generate loss-of-function mutants and gene overexpression in stable transgenic lines, complemented by the development of near-isogenic lines (NILs) to dissect the genetic effect of each QTL in a uniform background.

The major QTL *QGPC.lfnu-4B*, which explained over 11% of phenotypic variance and was consistently detected across all four environments, did not yield qualified candidate genes under our current screening criteria, yet it remains an exceptionally valuable locus. This region warrants fine-mapping using high-resolution recombinant populations to narrow the candidate interval and ultimately clone the causal gene(s). Furthermore, haplotype mining in diverse natural populations and transcriptomic profiling during grain filling would help elucidate the regulatory networks and expression dynamics of these candidate genes. Integrating multi-omics approaches, transcriptomics, proteomics, and metabolomics, could reveal the metabolic pathways through which these genes modulate GPC at a systems level. Ultimately, understanding the functional mechanisms and regulatory networks of these candidate genes will enable the development of wheat varieties with improved GPC without compromising yield. The stable QTLs and validated KASP markers reported here provide essential resources and tools for the molecular breeding of wheat varieties with superior grain protein content.

## 4. Materials and Methods

### 4.1. Plant Materials

A total of 262 F_5_ RILs derived from the cross between Linmai2 and Zhong892 were used for QTL mapping. A diverse panel of 140 wheat cultivars was used to validate the effects of identified QTL. The Linmai2/Zhong892 RIL population along with the parental lines were evaluated across four environments during the 2020–2021 and 2021–2022 cropping seasons at Xinxiang in Henan Province, and Dezhou in Shandong Province, e.g., Xinxiang2021 (E1), Xinxiang2022 (E2), Dezhou2021 (E3) and Dezhou2022 (E4). Field trials were arranged in a randomized complete block design with three replications. Each line was planted in three row plots of 1.5 m in length, with 25 cm between rows and 50 seeds per row. All field trials followed local standard management practices. The BLUE values were additionally calculated to eliminate environmental noise for consensus QTL detection. The field experiments for the 140 wheat cultivars were conducted over two consecutive growing seasons (2022–2023 and 2023–2024) at the Zhumadian in Henan (114.02° E, 32.59° N) and the Yangling in Shaanxi (108.04° E, 34.70° N), with three randomized replicates per cultivar in each location–year environment. After harvest, grains were naturally sun-dried and stored, and GPC was measured by Swiss-made near-infrared quality analyzer (DA7200). The instrument was pre-calibrated with 200 wheat grain samples covering a GPC range of 10.0–19.5% with reference chemical Kjeldahl measurements; the measurement repeatability error was less than 0.15% GPC. The BLUE GPC values were used for subsequent t-test association analysis between KASP genotypes and grain protein content (Table 6, Table A3).

### 4.2. Genotyping for the Linmai2/Zhong892 RIL Population

Genomic DNA was extracted from young leaves following the CTAB method described by Porebski et al. [27]. In this study, SNP genotyping of the two parental lines (Linmai2, Zhong892) and all 262 RIL lines was conducted using the wheat Illumina 90K SNP array (Capital-Bio, Beijing, China). A total of 80,587 raw SNP markers were obtained from chip scanning. The high-density genetic linkage map constructed based on this SNP dataset was previously published by Liu et al. [28], and we directly adopted this published genetic map framework for subsequent GPC QTL linkage. Inclusive composite interval mapping (ICIM) in IciMapping v4.1 was employed for QTL mapping of GPC in the Linmai2/Zhong892 RIL population across four environments [29]. This approach integrates interval mapping with multiple regression analysis. A genome-wide LOD threshold of 2.72 was determined via 1000 permutation tests at *p* = 0.05; QTLs with LOD scores above this threshold were retained for further analysis. A QTL was defined as stable if it was consistently detected in at least two environments with similar additive effect directions, following the widely adopted criteria in recent QTL mapping studies. Physical positions of mapped SNPs were obtained by blasting the flanking sequences against the Chinese Spring reference genome (IWGSC v1.0).

### 4.3. Conversion of SNPs to KASP Markers

KASP markers were developed from array-based SNP markers tightly linked to stable and effective loci within and flanking the identified GPC QTL. Primer design was performed using PolyMarker v1.1.5 (http://www.polymarker.info/ accessed on 22 July 2026; Ramirez-Gonzalez et al. [30]). Each KASP primer set consists of two competitive allele-specific primers (differing by a single base at the 3′ end) and one common reverse primer. Locus-specific or semi-specific primers were selected; the two competitive primers were appended with FAM and HEX tag sequences at their 5′ ends, respectively. All primer sequences were synthesized by Sangon Biotech (Shanghai, China) Co., Ltd. (Table A3).

KASP genotyping was carried out in a 4 μL reaction containing 2 μL 2× KASP Master Mix, 0.045 μL KASP primer mix, and 2 μL genomic DNA (30 ng/μL). Amplification was performed using a water-bath PCR instrument (LGC, London, UK) with the following thermal profile: 94 °C for 15 min (1 cycle); 10 cycles of 94 °C for 20 s and touchdown annealing from 65 °C to 57 °C for 1 min; then 35 cycles of 94 °C for 20 s and 57 °C for 1 min. Temperature and duration were adjusted as needed to ensure efficient amplification.

After PCR, the plate was scanned using the 384-KASP program on a PHERAstarplus SNP genotyping platform (LGC Science Shanghai Ltd., Shanghai, China) or a PHEstar instrument (BMG Labtech GmbH, Ortenberg, Germany), employing single excitation and dual emission light for fluorescence detection. Allele discrimination and signal scanning were performed automatically using KlusterCaller™ software (version 2.24.0.11, LGC, Hoddesdon, UK) or KLUSTER software (LGC, Hoddesdon, UK), without gel-based separation. Typically, samples with only FAM fluorescence emit blue signals, those with only HEX fluorescence emit red signals, and those with both emit green signals.

### 4.4. Statistical Analysis

ANOVA, phenotypic correlation coefficients, and Student’s t-tests were performed using SAS 9.2 (SAS Institute Inc., Cary, NC, USA). ANOVA was carried out with the PROC MIXED procedure to evaluate the contributions of genotype (lines) and environment, with genotype, environment, genotype by environment interaction, and replicate nested within environment all treated as random effects. *F*-values derived from ANOVA were marked with significance asterisks: *** represents *p* < 0.001, indicating extremely significant effects of corresponding variance sources on GPC. In parallel, a model with genotype considered as a fixed effect was fitted to obtain best linear unbiased estimates (BLUEs) of genotype performance across environments. Adjusted means for GPC in individual environments and across environments were separately estimated using PROC MIXED. Broad sense heritability (*H*_b_^2^) was calculated as: *H*_b_^2^ = σg^2^/(σg^2^ + σge^2^/r + σε^2^/(r·e)). where σg^2^, σge^2^, and σε^2^ represent the variance components for genotype, genotype by environment interaction, and residual error, respectively, while e and r denote the number of environments and the number of replicates within each environment.

To validate QTL effects, differences in GPC between the two homozygous genotype classes (Linmai2 and Zhong892 types) were assessed. For the progeny tests, phenotypic differences in GPC between the two homozygous genotype groups were evaluated using Student’s *t*-tests.

### 4.5. Candidate Gene Prediction

Candidate genes were selected using three sequential, clearly defined screening criteria. First, all high-confidence gene models fully contained within the physical start–end intervals of each stable GPC QTL were extracted based on IWGSC RefSeq v1.0 annotation, while genes extending beyond the QTL boundaries were excluded; the resulting candidate gene list is provided in Table A2. Second, only genes annotated to core biological pathways that directly regulate grain protein accumulation were retained, including nitrogen assimilation, asparagine/amino acid biosynthesis, amino acid transmembrane transport, intracellular protein turnover, and carbon–nitrogen balance, given that nitrogen remobilization from source leaves to the developing endosperm, amino acid translocation into grain cells, and storage protein synthesis represent the three rate-limiting steps determining final GPC and mutations or expression variation in these genes directly alter grain nitrogen deposition efficiency; genes associated with cell development, disease resistance, photosynthesis, or secondary metabolism were discarded unless they possessed a clearly defined nitrogen regulatory function. Third, gene expression values were queried against the expVIP wheat public expression database, and only genes with moderate or high expression in both flag leaves (the source tissue for nitrogen remobilization) and developing grains (the sink tissue for protein storage) were retained; genes with extremely low or undetectable expression in either tissue were excluded, even if annotated to nitrogen-related pathways. All genes simultaneously satisfying these three criteria were regarded as prioritized candidates for subsequent functional analysis.

## 5. Conclusions

In this study, five QTLs associated with GPC were identified in a Linmai2/Zhong892 RIL population, individually explaining 5.21% to 11.38% of the phenotypic variance. Four breeder-friendly KASP markers targeting the major QTLs were developed and validated, each showing highly significant differentiation between high- and low-GPC alleles, thereby demonstrating their potential for marker-assisted selection. Additionally, twelve candidate genes involved in nitrogen assimilation, amino acid biosynthesis and transport, and protein turnover were prioritized within the QTL intervals, providing plausible biological links to GPC regulation. Future efforts should focus on fine mapping the major QTL, functional characterization of the candidate genes, and evaluating the pyramiding effects across diverse genetic backgrounds and environments. This study provides essential resources and tools for the molecular breeding of wheat varieties with superior grain protein content.

## Figures and Tables

**Table 1:** The summary of the details for grain protein content in the Linmai2/Zhong892 RIL population.

Environment	E1 (%)	E2 (%)	E3 (%)	E4 (%)
Zhong892 (high GPC)	15.72	15.96	15.68	15.85
Linmai2 (low GPC)	11.41	11.28	11.35	11.47
Max	18.83	19.23	19.17	19.10
Min	10.30	10.33	10.30	10.40
Mean	13.28	13.30	13.22	13.33
Std	2.30	2.35	2.22	2.38
CV	0.17	0.18	0.17	0.18

E1: Xinxiang2021, E2: Xinxiang2022, E3: Dezhou2021 and E4 Dezhou2022.

**Table 2:** ANOVA for the grain protein content in the Linmai2/Zhong892 RIL population.

Source of Variation	DF	Sum of Square	Mean Square	F Value	Significance	Variance Component
Environment	8	3.66	0.4575	3.9931	***	0.0013
Genotype	261	2153.79	8.2521	72.0211	***	0.6781
Genotype × Environment	3	133.99	44.6636	389.8065	***	0.0567
Replicate (Environment)	783	563.09	0.7192	6.2765	***	0.2015

*** Indicates significant at the *p* < 0.0001 level.

**Table 3:** Linkage mapping for the grain protein content in the Linmai2/Zhong892 RIL population.

QTL	Env.	Chr.	Left Marker	Right Marker	Interval (Mb)	2-LOD Interval (Mb)	LOD	PVE (%)	Add
*QGPC.lfnu-1BL*	E1/E2	1B	*wsnp_CAP11_rep_c4138_1957470*	*BS00000010_51*	668.0–670.8	668.3–670.4	2.89–3.68	6.12–6.65	0.6–0.7
*QGPC.lfnu-2BS*	E1/E2/E3/BLUE	2B	*Ra_c11464_294*	*Kukri_rep_c113048_451*	1.3–6.9	1.9–6.2	2.89–3.20	5.62–5.99	−0.5–0.6
*QGPC.lfnu-4AS*	E2/E3	4A	*RAC875_c65408_104*	*Tdurum_contig10595_633*	9.1–13.7	9.5–13.2	2.86–3.05	5.21–5.78	0.7–0.8
*QGPC.lfnu-4B*	E1/E2/E3/E4/BLUE	4B	*Kukri_rep_c107274_497*	*Excalibur_c60276_110*	285.4–290.2	286.3–289.3	5.20–8.82	7.63–11.38	1.0–1.12
*QGPC.lfnu-6BS*	E2/E4/BLUE	6B	*Tdurum_contig45478_176*	*Kukri_c11002_927*	97.8–102.1	98.3–101.6	3.85–4.26	6.21–7.25	0.7–0.8

E1: Xinxiang2021, E2: Xinxiang2022, E3: Dezhou2021 and E4 Dezhou2022.

**Table 4:** The candidate genes for the loci identified in this study.

QTL	Candidate Gene ID	Chr.	Physical Interval (bp)	Functional Annotation	Proposed Mechanism
*QGPC.lfnu-1BL*	*TraesCS1B01G449900*	1B	668,182,903–668,184,142	4-hydroxy-tetrahydrodipicolinate synthase	Lysine biosynthesis, affecting amino acid pool
*QGPC.lfnu-1BL*	*TraesCS1B01G452800*	1B	669,461,869–669,462,231	Carboxypeptidase	Protein degradation and turnover rate regulation
*QGPC.lfnu-1BL*	*TraesCS1B01G453600*	1B	669,754,736–669,761,058	Asparagine synthetase	Nitrogen transport and remobilization, providing nitrogen carrier
*QGPC.lfnu-2BS*	*TraesCS2B01G251700*	2B	265,215,556–265,220,140	NPF transporter	Transmembrane transport of amino acids into grains
*QGPC.lfnu-4AS*	*TraesCS4A01G016900*	4A	11,364,323–11,367,895	3-dehydroquinate synthase	Shikimate pathway, aromatic amino acid synthesis
*QGPC.lfnu-4AS*	*TraesCS4A01G018300*	4A	11,841,295–11,841,885	Amino acid transporter-like	Transmembrane transport of amino acids into grains
*QGPC.lfnu-4AS*	*TraesCS4A01G018400*	4A	11,987,704–11,988,999	Amino acid transporter-like	Transmembrane transport of amino acids into grains
*QGPC.lfnu-4AS*	*TraesCS4A01G018500*	4A	12,153,638–12,154,933	Amino acid transporter-like	Transmembrane transport of amino acids into grains
*QGPC.lfnu-4AS*	*TraesCS4A01G015900*	4A	9,929,288–9,932,731	Aminotransferase like protein	Conversion of nitrogen intermediates, affecting amino acid composition
*QGPC.lfnu-4B*	*TraesCS4B01G156000*	4B	285,530,769–285,580,442	E3 ubiquitin protein ligase	Conversion of nitrogen intermediates, affecting amino acid composition
*QGPC.lfnu-4B*	*TraesCS4B01G156200*	4B	286,067,795–286,073,022	PPR protein/GR-RBP	Conversion of nitrogen intermediates, affecting amino acid composition
*QGPC.lfnu-6BS*	*TraesCS6B01G116400*	6B	101,917,058–101,922,889	Beta-amylase	Carbon-nitrogen balance, altering source-sink relationship

**Table 5:** The KASP marker primer sequences identified in this study.

QTL	KASP Marker	Allele	FAM	HEX	Common
*QGPC.lfnu-1BL*	*KASP-GPC-1BL*	T/C	caatggacaatgaatgatacacacT	caatggacaatgaatgatacacacC	tgcttccagttccatcgatctatgC
*QGPC.lfnu-2BS*	*KASP-GPC-2BS*	A/G	gccacgcctcgtAaTtatcTcA	gccacgcctcgtAaTtatcTcG	GgagacgggttgAcctGgaA
*QGPC.lfnu-4B*	*KASP-GPC-4B*	T/C	gctCAgttgcgccatctA	gctCAgttgcgccatctG	cgtgactcacagattctaccaaaac
*QGPC.lfnu-6BS*	*KASP-GPC-6BS*	T/C	ggtCagacacagagcGacA	ggtCagacacagagcGacG	ggaagaagtcCtcggctcgG

**Table 6:** The KASP markers validated in the 140 wheat accessions.

KASP Marker	QTL	Allele	Number of Varieties	Grain Protein Content (%)	Effect Size (Mean Difference, %)	Standard Error (SE)	95% Confidence Interval	R^2^ (% Variance Explained)	*p* Value
*KASP-GPC-1BL*	*QGPC.lfnu-1BL*	TT	66	14.5	0.807	0.145	0.519–1.094	18.27	*p* < 0.05
		CC	74	15.0					
*KASP-GPC-2BS*	*QGPC.lfnu-2BS*	AA	65	15.0	0.445	0.156	0.136–0.754	5.55	*p* < 0.05
		GG	75	14.4					
*KASP-GPC-4B*	*QGPC.lfnu-4B*	TT	79	14.5	0.700	0.150	0.403–0.997	13.58	*p* < 0.05
		CC	61	15.0					
*KASP-GPC-6BS*	*QGPC.lfnu-6BS*	TT	70	14.4	0.679	0.150	0.382–0.975	12.93	*p* < 0.05
		CC	70	15.1					

## Data Availability

All of the data generated or analyzed during this study are included in this published article.

## Abbreviations

The following abbreviations are used in this manuscript:

KASP	competitive allele-specific PCR
GPC	grain protein content
WGC	wet gluten content
QTL	quantitative trait loci
SNP	single nucleotide polymorphism

## Appendix A

**Table A1:** The reported GPC loci and QTL identified in this study.

Chr.	Locus or Gene	Physical Location (Mb)	References
1B	*QGPC.lfnu-1BL*	668.0–670.8	This study
1B	*qGPC.1B.1*	Unknown, gwm413–cfd65	[21]
1B	*QGpc.sdau-1B*	Unknown	[48]
1B	*QGpc.iari_1B*	Unknown	-
2B	*QGPC.lfnu-2BS*	1.3–6.9	This study
2B	*QGpc.2B-yume*	Unknown (Xgpw4382)	[49]
2B	*QGpc.cd1-2B.1*	Unknown (3.93–8.70 cM)	[50]
2B	*QGpc.ipk-2B*	658–674	-
4A	*QGPC.lfnu-4AS*	9.1–13.7	This study
4A	*QGpc.sdau-4A*	Unknown	[48]
4A	*QGpc.his-4A-2*	629.36–701.53	-
4A	*QGpc.iari_4A*	Unknown	-
4B	*QGPC.lfnu-4B*	285.4–290.2	This study
4B	*QGpc.yaas-4B*	Co-located with Rht-B1	[51]
4B	*QQrr.cau-4B*	Unknown	-
4B	*QGpc.iari_4B*	Unknown (Xgwm149–AX-94559916)	-
6B	*QGPC.lfnu-6BS*	97.8–102.1	This study
6B	*Gpc-B1 (NAM-B1)*	99.1–99.5	[12]
6B	*QGpc.ndsu-6Bb*	Unknown (centromere; Xabg387-6B)	-
6B	*DIC-6B locus*	Unknown (centromere and Nor-B2)	[52]
6B	*GPC-6B1*	Unknown (Xcdo365)	[34]

**Table A2:** The Grain protein content and KASP marker genotype data in the natural population.

Gene	Chr.	Start (bp)	End (bp)	Annotation
*TraesCS1B01G447600*	1B	667,224,751	667,229,526	Zinc finger CCCH domain protein
*TraesCS1B01G447700*	1B	667,262,240	667,264,084	Methionyl-tRNA formyltransferase
*TraesCS1B01G447800*	1B	667,306,894	667,308,119	DUF1645 family protein
*TraesCS1B01G447900*	1B	667,423,345	667,428,725	Cinnamoyl-CoA reductase
*TraesCS1B01G448000*	1B	667,487,816	667,490,349	Glutamyl-tRNA (Gln) amidotransferase subunit A
*TraesCS1B01G448100*	1B	667,491,123	667,494,420	Polygalacturonase
*TraesCS1B01G448200*	1B	667,620,091	667,620,831	GATA transcription factor, putative
*TraesCS1B01G448300*	1B	667,677,935	667,682,146	Sugar transporter protein
*TraesCS1B01G448400*	1B	667,716,006	667,716,995	DNA-3-methyladenine glycosylase 1
*TraesCS1B01G448500*	1B	667,737,946	667,739,895	Acyl-CoA ligase-like
*TraesCS1B01G448600*	1B	667,749,720	667,750,478	Zinc finger homeodomain protein 1
*TraesCS1B01G448700*	1B	667,819,823	667,823,125	Receptor-like protein kinase
*TraesCS1B01G448800*	1B	667,824,521	667,825,040	Endo-1,4-beta-xylanase 2
*TraesCS1B01G448900*	1B	667,831,048	667,832,748	FAD-binding Berberine family protein
*TraesCS1B01G449000*	1B	667,918,106	667,918,611	RADIALIS-like transcription factor
*TraesCS1B01G449100*	1B	667,930,932	667,931,301	Transcription factor radialis-like protein
*TraesCS1B01G449200*	1B	667,965,704	667,966,088	RADIALIS-like transcription factor
*TraesCS1B01G449300*	1B	667,996,876	667,997,245	RADIALIS-like transcription factor
*TraesCS1B01G449400*	1B	668,030,249	668,031,183	RADIALIS-like transcription factor
*TraesCS1B01G449500*	1B	668,053,694	668,056,372	Anaphase-promoting complex subunit 10
*TraesCS1B01G449600*	1B	668,118,006	668,126,459	Auxilin-like protein 1
*TraesCS1B01G449700*	1B	668,129,122	668,132,472	Glucose-1-phosphate adenylyltransferase
*TraesCS1B01G449800*	1B	668,140,213	668,143,984	Rad3-related DNA helicase family protein
*TraesCS1B01G449900*	1B	668,182,997	668,183,939	4-hydroxy-tetrahydrodipicolinate synthase
*TraesCS1B01G450000*	1B	668,186,403	668,187,327	Methyltransferase
*TraesCS1B01G450100*	1B	668,199,849	668,201,062	Auxin efflux carrier family protein
*TraesCS1B01G450200*	1B	668,436,557	668,437,003	Multidrug resistance protein MdtC
*TraesCS1B01G450300*	1B	668,468,337	668,472,200	cyclic nucleotide-gated channel 6
*TraesCS1B01G450400*	1B	668,648,632	668,655,434	Sister chromatid cohesion 1 protein 1
*TraesCS1B01G450500*	1B	668,674,952	668,676,202	Transmembrane protein, putative
*TraesCS1B01G450600*	1B	668,715,022	668,718,328	Phosphatidate cytidylyltransferase, mitochondrial
*TraesCS1B01G450700*	1B	668,756,926	668,763,146	Kinase-related protein DUF1296
*TraesCS1B01G450800*	1B	668,805,723	668,805,977	BnaCnng55980D protein
*TraesCS1B01G450900*	1B	668,808,033	668,808,668	Ribosomal protein S1
*TraesCS1B01G451000*	1B	668,808,804	668,809,691	Cytochrome c biogenesis
*TraesCS1B01G451100*	1B	668,911,699	668,912,595	Chlorophyll a-b binding protein, chloroplastic
*TraesCS1B01G451200*	1B	668,911,879	668,912,613	phosphatidylinositol-4-phosphate 5-kinase 2
*TraesCS1B01G451300*	1B	668,919,596	668,919,904	TTF-type zinc finger protein with HAT dimerization domain-containing protein
*TraesCS1B01G451400*	1B	668,922,442	668,922,858	magnesium transporter 7
*TraesCS1B01G451500*	1B	668,936,376	668,938,364	Chaperone protein DnaJ
*TraesCS1B01G451600*	1B	668,960,976	668,963,003	Receptor-like protein kinase
*TraesCS1B01G451700*	1B	668,966,419	668,968,483	receptor kinase 1
*TraesCS1B01G451800*	1B	668,982,087	668,983,260	Glucan endo-1,3-beta-glucosidase 1
*TraesCS1B01G451900*	1B	669,022,511	669,023,410	Plant basic secretory protein family protein, putative
*TraesCS1B01G452000*	1B	669,027,373	669,033,817	CAS1 domain-containing protein 1
*TraesCS1B01G452100*	1B	669,151,016	669,153,958	Solanesyl diphosphate synthase family protein
*TraesCS1B01G452200*	1B	669,158,085	669,163,178	Mitogen-activated protein kinase
*TraesCS1B01G452300*	1B	669,191,801	669,192,428	Phytochrome interacting factor 3, putative isoform 3
*TraesCS1B01G452400*	1B	669,219,295	669,220,671	Carboxypeptidase
*TraesCS1B01G452500*	1B	669,223,789	669,224,860	Chymotrypsin inhibitor
*TraesCS1B01G452600*	1B	669,254,378	669,254,860	Disease resistance protein (CC-NBS-LRR class) family
*TraesCS1B01G452700*	1B	669,336,271	669,336,588	SPT2 chromatin protein
*TraesCS1B01G452800*	1B	669,461,869	669,462,231	Carboxypeptidase
*TraesCS1B01G452900*	1B	669,643,650	669,652,979	Ta11-like non-LTR retrotransposon
*TraesCS1B01G453000*	1B	669,658,138	669,659,664	transmembrane protein, putative (DUF247)
*TraesCS1B01G453100*	1B	669,705,594	669,707,036	ADP-ribosylation factor
*TraesCS1B01G453200*	1B	669,709,450	669,713,975	WRKY transcription factor
*TraesCS1B01G453300*	1B	669,728,850	669,731,151	WRKY transcription factor
*TraesCS1B01G453400*	1B	669,748,178	669,750,400	Subtilisin-like protease
*TraesCS1B01G453500*	1B	669,751,414	669,751,683	Mitochondrial import receptor subunit TOM7-1
*TraesCS1B01G453600*	1B	669,755,028	669,760,792	Asparagine synthetase
*TraesCS1B01G453700*	1B	669,895,926	669,902,224	Sorting nexin-16
*TraesCS1B01G453800*	1B	669,919,193	669,919,819	F-box family protein
*TraesCS1B01G453900*	1B	669,922,599	669,924,501	Glucan endo-1,3-beta-glucosidase 3
*TraesCS1B01G454000*	1B	670,025,374	670,028,845	Receptor-like protein kinase, putative, expressed
*TraesCS1B01G454100*	1B	670,034,275	670,036,954	Receptor-like protein kinase, putative, expressed
*TraesCS1B01G454200*	1B	670,142,456	670,143,972	Glucan endo-1,3-beta-glucosidase 3
*TraesCS1B01G454300*	1B	670,145,227	670,148,068	BED zinc finger, hAT family dimerization domain
*TraesCS1B01G454400*	1B	670,153,097	670,155,861	Receptor-like protein kinase, putative, expressed
*TraesCS1B01G454500*	1B	670,158,940	670,160,833	Glucan endo-1,3-beta-glucosidase 3
*TraesCS1B01G454600*	1B	670,165,114	670,167,686	Receptor-like protein kinase, putative, expressed
*TraesCS1B01G454700*	1B	670,170,578	670,173,316	Receptor-like protein kinase, putative, expressed
*TraesCS1B01G454800*	1B	670,180,418	670,187,792	Sugar transporter, putative
*TraesCS1B01G454900*	1B	670,181,369	670,183,761	Protein kinase
*TraesCS1B01G455000*	1B	670,202,262	670,203,245	WRKY family transcription factor
*TraesCS1B01G455200*	1B	670,384,217	670,389,742	ATP-dependent zinc metalloprotease FtsH 1
*TraesCS1B01G455300*	1B	670,395,914	670,402,241	Carboxypeptidase
*TraesCS1B01G455400*	1B	670,570,256	670,571,017	membrane-associated kinase regulator
*TraesCS1B01G455500*	1B	670,673,389	670,673,712	RING/FYVE/PHD zinc finger superfamily protein
*TraesCS1B01G455600*	1B	670,675,142	670,675,480	Peptide chain release factor 1
*TraesCS1B01G455700*	1B	670,714,557	670,715,318	Late embryogenesis abundant hydroxyproline-rich glycoprotein family, putative
*TraesCS1B01G455800*	1B	670,743,638	670,745,203	F-box family protein
*TraesCS1B01G455900*	1B	670,783,621	670,784,204	Late embryogenesis abundant protein Lea14
*TraesCS1B01G456600*	1B	671,196,273	671,198,784	Protein ABIL1
*TraesCS1B01G456700*	1B	671,199,679	671,202,696	Calcium-dependent protein kinase
*TraesCS2B01G002100*	2B	1,348,975	1,361,789	Inosine-5′-monophosphate dehydrogenase
*TraesCS2B01G002200*	2B	1,386,371	1,395,906	receptor kinase 1
*TraesCS2B01G002300*	2B	1,452,850	1,453,224	Ribosome maturation factor RimP
*TraesCS2B01G002400*	2B	1,499,379	1,499,731	Neuraminidase
*TraesCS2B01G002500*	2B	1,520,221	1,522,260	transmembrane protein, putative (DUF594)
*TraesCS2B01G002600*	2B	1,600,742	1,601,266	Specific tissue protein
*TraesCS2B01G002700*	2B	1,706,416	1,706,988	ABA-responsive binding factor
*TraesCS2B01G002800*	2B	1,749,609	1,750,895	Sulfotransferase
*TraesCS2B01G003100*	2B	1,764,773	1,766,058	Sulfotransferase
*TraesCS2B01G003600*	2B	1,831,881	1,833,776	FCH domain only protein 1
*TraesCS2B01G003800*	2B	2,089,681	2,091,723	BTB/POZ domain-containing protein kctd15
*TraesCS2B01G003900*	2B	2,107,170	2,108,718	Cleavage stimulation factor subunit 1
*TraesCS2B01G004000*	2B	2,109,765	2,112,796	Fructokinase-2
*TraesCS2B01G004100*	2B	2,116,046	2,116,633	Alpha amylase inhibitor protein
*TraesCS2B01G004300*	2B	2,335,867	2,336,235	Defensin
*TraesCS2B01G004800*	2B	2,566,039	2,566,626	Alpha amylase inhibitor protein
*TraesCS2B01G004900*	2B	2,639,641	2,643,293	Kinase
*TraesCS2B01G005000*	2B	3,288,914	3,292,687	Receptor protein kinase
*TraesCS2B01G005100*	2B	3,300,441	3,306,813	AAR2 family protein
*TraesCS2B01G005200*	2B	3,307,731	3,312,736	Cell differentiation protein RCD1
*TraesCS2B01G005300*	2B	3,398,345	3,406,093	Sterol 3-beta-glucosyltransferase
*TraesCS2B01G005400*	2B	3,407,138	3,410,526	Receptor-kinase, putative
*TraesCS2B01G005500*	2B	3,659,755	3,661,311	Leucine-rich repeat receptor-like protein kinase family protein
*TraesCS2B01G005600*	2B	3,684,728	3,687,040	Leucine-rich repeat receptor-like protein kinase family protein
*TraesCS2B01G005700*	2B	3,687,109	3,687,952	Protein kinase family protein
*TraesCS2B01G006000*	2B	3,794,600	3,795,885	Chalcone synthase
*TraesCS2B01G006100*	2B	3,839,152	3,840,387	Alpha/beta-Hydrolases superfamily protein
*TraesCS2B01G006300*	2B	4,013,566	4,015,769	Chalcone synthase
*TraesCS2B01G006400*	2B	4,117,048	4,118,042	Heptahelical transmembrane protein 4
*TraesCS2B01G006500*	2B	4,131,505	4,132,740	Alpha/beta-Hydrolases superfamily protein
*TraesCS2B01G006700*	2B	4,283,163	4,285,479	Chalcone synthase
*TraesCS2B01G006800*	2B	4,425,195	4,426,546	Chalcone synthase
*TraesCS2B01G007000*	2B	4,482,082	4,483,377	Chalcone synthase
*TraesCS2B01G007100*	2B	4,493,310	4,494,653	Alpha/beta-Hydrolases superfamily protein
*TraesCS2B01G007200*	2B	4,502,972	4,515,019	O-acyltransferase WSD1
*TraesCS2B01G007300*	2B	4,622,267	4,624,951	Ankyrin repeat-containing protein
*TraesCS2B01G007400*	2B	4,633,720	4,637,892	Lectin-related protein
*TraesCS2B01G007500*	2B	4,642,754	4,643,683	NBS-LRR disease resistance protein, putative
*TraesCS2B01G007600*	2B	4,644,215	4,645,528	NBS-LRR disease resistance protein, putative, expressed
*TraesCS2B01G007700*	2B	4,646,830	4,653,489	Protein DA1-related 1
*TraesCS2B01G007800*	2B	4,656,500	4,665,574	Serine-rich 25 kDa antigen protein
*TraesCS2B01G007900*	2B	4,714,311	4,715,453	Protein PLANT CADMIUM RESISTANCE 2
*TraesCS2B01G008000*	2B	4,720,640	4,721,573	Protein PLANT CADMIUM RESISTANCE 2
*TraesCS2B01G008100*	2B	4,727,007	4,728,192	Protein PLANT CADMIUM RESISTANCE 2
*TraesCS2B01G008200*	2B	4,740,306	4,740,854	DNA topoisomerase
*TraesCS2B01G008300*	2B	4,751,819	4,754,293	Serine/threonine-protein kinase
*TraesCS2B01G008400*	2B	4,766,106	4,771,497	Protein kinase
*TraesCS2B01G008500*	2B	4,790,073	4,794,339	Ankyrin repeat protein family-like protein
*TraesCS2B01G008700*	2B	4,882,497	4,887,091	Receptor-kinase, putative
*TraesCS2B01G008800*	2B	4,923,626	4,924,189	Rho GTPase activation protein (RhoGAP) with PH domain-containing protein
*TraesCS2B01G008900*	2B	4,984,390	4,990,447	Protein transport Sec24-like protein
*TraesCS2B01G009000*	2B	5,010,495	5,010,806	SNF2 domain-containing protein/helicase domain-containing protein/zinc finger protein-like protein
*TraesCS2B01G009200*	2B	5,118,894	5,119,436	Ribosomal protein L11 methyltransferase
*TraesCS2B01G009300*	2B	5,370,680	5,373,104	Bidirectional sugar transporter SWEET
*TraesCS2B01G009400*	2B	5,430,438	5,430,752	SNF2 domain-containing protein/helicase domain-containing protein/zinc finger protein-like protein
*TraesCS2B01G009500*	2B	5,479,314	5,483,429	Cytochrome oxidase complex assembly protein
*TraesCS2B01G009600*	2B	5,579,177	5,580,297	cytochrome oxidase complex assembly protein
*TraesCS2B01G009700*	2B	5,592,854	5,593,323	Maternally-expressed gene (MEG)/Ae1-like cysteine-rich peptide
*TraesCS2B01G009800*	2B	5,596,473	5,596,973	Fatty acyl-CoA reductase 2
*TraesCS2B01G009900*	2B	5,603,155	5,604,399	Fanconi anemia group I-like protein
*TraesCS2B01G010000*	2B	5,608,439	5,610,429	Werner Syndrome-like exonuclease
*TraesCS2B01G010100*	2B	5,628,353	5,629,618	Flavin-containing monooxygenase
*TraesCS2B01G010200*	2B	5,661,615	5,667,191	Evolutionarily conserved C-terminal region 2
*TraesCS2B01G010300*	2B	5,669,529	5,673,046	DUF1644 family protein
*TraesCS2B01G010400*	2B	5,678,113	5,681,786	TOM1-like protein 2
*TraesCS2B01G010500*	2B	5,684,865	5,689,380	WRKY transcription factor
*TraesCS2B01G010700*	2B	5,785,090	5,786,189	Leucine-rich repeat receptor-like kinase
*TraesCS2B01G010800*	2B	5,796,719	5,807,192	Mediator of RNA polymerase II transcription subunit 15a
*TraesCS2B01G010900*	2B	5,822,681	5,824,899	Agenet domain, putative
*TraesCS2B01G011000*	2B	5,828,065	5,828,772	Beige/BEACH and WD40 domain-containing protein
*TraesCS2B01G011100*	2B	5,993,745	5,998,277	Gibberellin-regulated family protein
*TraesCS2B01G011200*	2B	6,004,747	6,006,538	Leucine-rich repeat receptor-like kinase
*TraesCS2B01G011300*	2B	6,022,757	6,024,383	Leucine-rich repeat receptor-like kinase
*TraesCS2B01G011400*	2B	60,35,821	6,039,223	Receptor protein kinase, putative
*TraesCS2B01G011500*	2B	6,057,152	6,059,629	Leucine-rich repeat receptor-like protein kinase family protein, putative
*TraesCS2B01G011600*	2B	6,069,418	6,074,804	NBS-LRR disease resistance protein
*TraesCS2B01G011700*	2B	6,132,796	6,133,192	Gibberellin-regulated protein 1
*TraesCS2B01G011800*	2B	6,136,621	6,139,962	Receptor protein kinase, putative
*TraesCS2B01G011900*	2B	6,156,191	6,159,919	Receptor protein kinase, putative
*TraesCS2B01G012000*	2B	6,178,582	6,180,252	Glycosyltransferase
*TraesCS2B01G012100*	2B	6,180,152	6,195,734	ABC transporter B family-like protein
*TraesCS2B01G012200*	2B	6,199,281	6,204,169	ABC transporter B family-like protein
*TraesCS2B01G012300*	2B	6,210,160	6,226,436	ABC transporter B family-like protein
*TraesCS2B01G012400*	2B	6,253,187	6,253,913	Avr9/Cf-9 rapidly elicited protein
*TraesCS2B01G012500*	2B	6,257,402	6,261,010	Pyruvate dehydrogenase E1 component subunit alpha
*TraesCS2B01G012600*	2B	6,263,090	6,265,566	StAR-related lipid transfer protein
*TraesCS2B01G012700*	2B	62,95,167	6,298,747	Homeobox leucine zipper protein
*TraesCS2B01G012800*	2B	6,307,617	6,309,289	Peptidyl-prolyl cis-trans isomerase
*TraesCS2B01G012900*	2B	6,310,993	6,316,291	tRNA-dihydrouridine(47) synthase [NAD(P)(+)]
*TraesCS2B01G013000*	2B	6,336,890	6,340,208	Peptide transporter
*TraesCS2B01G013100*	2B	6,340,410	6,341,900	Glycosyltransferase
*TraesCS2B01G013200*	2B	6,376,705	6,383,049	Terpene synthase
*TraesCS2B01G013300*	2B	6,405,872	6,412,446	Terpene synthase
*TraesCS2B01G013400*	2B	6,426,314	6,426,511	Terpene synthase
*TraesCS2B01G013600*	2B	6,718,287	6,719,786	ATP synthase E chain
*TraesCS2B01G013900*	2B	6,749,404	6,750,660	3′-N-debenzoyl-2′-deoxytaxol N-benzoyltransferase
*TraesCS2B01G014100*	2B	6,791,726	6,793,074	3′-N-debenzoyl-2′-deoxytaxol N-benzoyltransferase
*TraesCS4A01G014300*	4A	9,056,551	9,059,637	BURP domain protein RD22
*TraesCS4A01G014400*	4A	9,374,674	9,375,054	Cysteine proteinase inhibitor
*TraesCS4A01G014500*	4A	9,378,070	9,381,339	arginine/glutamate-rich 1 protein
*TraesCS4A01G014600*	4A	9,396,879	9,399,382	UDP-N-acetylglucosamine—N-acetylmuramyl-(pentapeptide) pyrophosphoryl-undecaprenol N-acetylglucosamine transferase
*TraesCS4A01G014700*	4A	9,449,269	9,451,501	Galactose oxidase/kelch repeat superfamily protein
*TraesCS4A01G014800*	4A	9,453,051	9,455,222	ARM repeat superfamily protein
*TraesCS4A01G014900*	4A	9,457,044	9,457,406	Cysteine proteinase inhibitor
*TraesCS4A01G015000*	4A	9,467,889	9,468,401	Glutamyl-tRNA(Gln) amidotransferase subunit A
*TraesCS4A01G015100*	4A	9,484,293	9,484,652	Cysteine proteinase inhibitor
*TraesCS4A01G015200*	4A	9,550,215	9,550,577	Cysteine proteinase inhibitor
*TraesCS4A01G015300*	4A	9,555,089	9,555,646	Cysteine proteinase inhibitor
*TraesCS4A01G015400*	4A	9,614,781	9,620,538	Calcium-binding protein 39, putative, expressed
*TraesCS4A01G015500*	4A	9,623,214	9,623,462	ARM repeat superfamily protein
*TraesCS4A01G015600*	4A	9,626,247	9,626,495	Ribosomal RNA large subunit methyltransferase K/L
*TraesCS4A01G015700*	4A	9,907,412	9,908,077	Protein translocase subunit SecA 2
*TraesCS4A01G015800*	4A	9,909,118	9,913,926	Nuclear pore complex protein NUP96
*TraesCS4A01G015900*	4A	9,929,567	9,932,449	Aminotransferase like protein
*TraesCS4A01G016000*	4A	10,164,603	10,165,067	Transcription factor
*TraesCS4A01G016100*	4A	10,534,235	10,534,555	splicing factor PWI domain-containing protein/RNA recognition motif (RRM)-containing protein
*TraesCS4A01G016200*	4A	10,637,275	10,638,570	Kelch repeat-containing protein
*TraesCS4A01G016300*	4A	10,938,614	10,939,218	Transcription factor protein
*TraesCS4A01G016400*	4A	11,183,282	11,188,151	Sucrose transporter
*TraesCS4A01G016500*	4A	11,272,872	11,273,864	Glutaredoxin family protein
*TraesCS4A01G016600*	4A	11,296,831	11,299,294	Homeobox protein, putative
*TraesCS4A01G016700*	4A	11,318,246	11,318,970	NAD(P)-linked oxidoreductase-like protein
*TraesCS4A01G016800*	4A	11,360,658	11,364,025	Protein kinase
*TraesCS4A01G017000*	4A	11,600,147	11,600,765	SAUR-like auxin-responsive family protein
*TraesCS4A01G017100*	4A	11,622,758	11,622,868	vacuolar ATP synthase catalytic subunit-related/V-ATPase-related/vacuolar proton pump-like protein
*TraesCS4A01G017200*	4A	11,646,568	11,648,337	RPM1-interacting protein 4
*TraesCS4A01G017300*	4A	11,671,310	11,675,641	Glycerol-3-phosphate dehydrogenase [NAD(P)+]
*TraesCS4A01G017400*	4A	11,676,825	11,678,425	Patatin
*TraesCS4A01G017500*	4A	11,706,010	11,707,099	ATP synthase subunit beta
*TraesCS4A01G017600*	4A	11,711,788	11,713,133	HNH endonuclease
*TraesCS4A01G017700*	4A	11,716,514	11,718,496	Dof zinc finger protein
*TraesCS4A01G017800*	4A	11,771,777	11,772,636	50S ribosomal L18-like protein
*TraesCS4A01G017900*	4A	11,774,572	11,776,119	Pentatricopeptide repeat-containing protein
*TraesCS4A01G018000*	4A	11,808,307	11,811,296	O-fucosyltransferase family protein
*TraesCS4A01G018100*	4A	11,811,770	11,813,302	Patatin
*TraesCS4A01G018200*	4A	11,816,609	11,817,489	Ripening related protein family
*TraesCS4A01G018300*	4A	11,841,262	11,844,782	Amino acid transporter-like
*TraesCS4A01G018400*	4A	11,987,704	11,988,999	Amino acid transporter-like
*TraesCS4A01G018500*	4A	12,153,546	12,155,051	Amino acid transporter-like
*TraesCS4A01G018600*	4A	12,161,978	12,163,036	F-box domain containing protein
*TraesCS4A01G018700*	4A	12,166,841	12,169,821	F-box and associated interaction domains-containing protein
*TraesCS4A01G018800*	4A	12,209,098	12,211,010	F-box family protein
*TraesCS4A01G018900*	4A	12,377,067	12,378,114	Protease inhibitor/seed storage/lipid transfer family protein
*TraesCS4A01G019000*	4A	12,392,763	12,395,030	50S ribosomal L32
*TraesCS4A01G019100*	4A	12,395,873	12,399,318	Ribulose-phosphate 3-epimerase
*TraesCS4A01G019200*	4A	12,400,417	12,402,312	F-box family protein
*TraesCS4A01G019300*	4A	12,598,877	12,600,490	Histone-lysine N-methyltransferase
*TraesCS4A01G019500*	4A	12,622,746	12,624,383	Pentatricopeptide repeat-containing protein
*TraesCS4A01G019600*	4A	12,681,076	12,685,003	Pentatricopeptide repeat-containing protein
*TraesCS4A01G019700*	4A	12,992,713	12,995,880	Pumilio
*TraesCS4A01G019800*	4A	13,654,580	13,655,716	DUF1997 family protein
*TraesCS4A01G019900*	4A	13,656,475	13,658,945	plant/protein
*TraesCS4A01G020000*	4A	13,742,607	13,744,304	Late embryogenesis abundant protein, expressed
*TraesCS4A01G020100*	4A	13,746,167	13,748,495	Major facilitator superfamily protein
*TraesCS4A01G020200*	4A	13,757,238	13,759,297	F-box/kelch-repeat protein
*TraesCS4A01G020300*	4A	13,775,329	13,775,619	Retrovirus-related Pol polyprotein from transposon 17.6
*TraesCS4A01G020400*	4A	14,020,487	14,025,068	Serine/threonine-protein phosphatase
*TraesCS4A01G020500*	4A	14,025,717	14,028,349	Fatty acyl-CoA reductase
*TraesCS4B01G156000*	4B	285,530,769	285,580,442	E3 ubiquitin-protein ligase RNF25
*TraesCS4B01G156100*	4B	285,602,866	285,622,479	Paladin
*TraesCS4B01G156200*	4B	286,068,167	286,072,985	glycine-rich RNA-binding-like protein
*TraesCS4B01G156300*	4B	286,073,247	286,074,902	Pentatricopeptide repeat-containing protein
*TraesCS4B01G156400*	4B	286,744,480	286,749,244	RNA-binding protein
*TraesCS4B01G156500*	4B	289,102,565	289,103,509	Mitochondrial transcription termination factor family protein
*TraesCS4B01G156600*	4B	291,841,661	291,843,303	Transposon protein, putative, Mutator sub-class
*TraesCS4B01G156700*	4B	291,843,456	291,844,611	Serine/threonine protein phosphatase 7 long form isogeny
*TraesCS4B01G156800*	4B	291,847,572	291,847,955	Oxidoreductase/ transition metal ion binding protein
*TraesCS4B01G156900*	4B	291,851,907	291,855,786	Peter Pan-like protein
*TraesCS4B01G157000*	4B	291,886,224	291,894,058	splicing factor-like protein
*TraesCS6B01G114700*	6B	97,816,249	97,819,115	RING/U-box superfamily protein
*TraesCS6B01G114800*	6B	98,853,332	98,856,607	E3 ubiquitin-protein ligase
*TraesCS6B01G114900*	6B	98,858,531	98,861,111	ADP-ribosylation factor
*TraesCS6B01G115000*	6B	98,983,896	98,987,304	Chaperone protein dnaJ
*TraesCS6B01G115100*	6B	98,992,452	98,992,979	Polyubiquitin
*TraesCS6B01G115200*	6B	100,288,571	100,290,842	Sodium Bile acid symporter family
*TraesCS6B01G115300*	6B	100,717,452	100,719,014	Myb/SANT-like DNA-binding domain protein
*TraesCS6B01G115400*	6B	100,752,355	100,761,714	Myb/SANT-like DNA-binding domain protein
*TraesCS6B01G115500*	6B	100,894,921	100,895,741	NADH dehydrogenase [ubiquinone] 1 beta subcomplex subunit 3-B
*TraesCS6B01G115600*	6B	100,951,973	100,954,159	transmembrane protein, putative (DUF594)
*TraesCS6B01G115700*	6B	100,957,028	100,958,119	Aspartate—tRNA(Asp/Asn) ligase
*TraesCS6B01G115800*	6B	101,166,988	101,167,606	Histone H2B
*TraesCS6B01G115900*	6B	101,222,151	101,222,789	Photosystem II 5 kDa protein, chloroplastic
*TraesCS6B01G116000*	6B	101,325,081	101,325,677	Histone H2B
*TraesCS6B01G116100*	6B	101,554,290	101,554,682	E3 ubiquitin-protein ligase MARCH6
*TraesCS6B01G116200*	6B	101,731,455	101,735,467	Protein LOW PSII ACCUMULATION 3, chloroplastic
*TraesCS6B01G116300*	6B	101,736,266	101,738,257	Pentatricopeptide repeat-containing protein
*TraesCS6B01G116400*	6B	101,917,131	101,922,548	Beta-amylase
*TraesCS6B01G116500*	6B	103,668,446	103,669,759	disease resistance family protein/LRR family protein

**Table A3:** The Grain protein content and KASP marker genotype data in the natural population.

Number	Name	GPC	KASP-GPC-1BL	KASP-GPC-2BS	KASP-GPC-4B	KASP-GPC-6BS
1	04Zhong36	15.2	TT	GG	CC	CC
2	19CA27	15.5	CC	GG	CC	CC
3	19CA97	16.1	TT	GG	CC	CC
4	Anke1704	15.2	CC	GG	TT	CC
5	Anke1901	13.9	TT	AA	TT	TT
6	Anke1908	12.4	TT	GG	TT	TT
7	Anke2008	14.4	TT	GG	CC	CC
8	Anke203	13.0	TT	AA	TT	CC
9	Anong1928	14.8	TT	GG	TT	TT
10	Bainong207	13.5	CC	GG	CC	CC
11	BainongChengzhu21	13.9	TT	GG	TT	CC
12	Baoliang5	15.1	TT	GG	CC	TT
13	Cunmai35	14.3	TT	AA	TT	TT
14	Dehongfumai21	15.5	TT	AA	CC	CC
15	Fanyuemai32	14.2	TT	AA	TT	TT
16	FengdeCunmai16	15.1	TT	GG	TT	TT
17	Fumai16	14.5	TT	GG	TT	TT
18	Fumai33	14.1	CC	GG	TT	TT
19	Guanmai13	14.0	CC	AA	TT	TT
20	Hengmai175364	13.9	TT	AA	TT	CC
21	Huacheng138	14.7	TT	GG	TT	CC
22	Huacheng865	13.8	TT	GG	CC	CC
23	Huaimai2118	14.5	TT	GG	TT	TT
24	Huaimai40	14.1	TT	GG	TT	TT
25	Huaiming36	14.8	TT	GG	CC	CC
26	Huamai17v65	13.7	TT	GG	TT	TT
27	Huamai2003	14.1	CC	AA	TT	TT
28	Huamai2100	14.6	TT	AA	CC	TT
29	Jiamai208	14.7	CC	AA	CC	TT
30	Jimai418	14.1	TT	GG	CC	CC
31	Jìmài44	15.3	CC	AA	CC	CC
32	Jinhedao17278	13.3	TT	GG	TT	TT
33	Jiyuanmai20	14.2	TT	AA	TT	CC
34	Kedaxia116	14.5	CC	AA	TT	CC
35	Lianmai1901	14.8	CC	GG	CC	CC
36	Liuxia526	14.4	CC	GG	TT	TT
37	Lunxuan148	16.2	CC	AA	CC	TT
38	Lunxuan369	14.4	CC	GG	TT	CC
39	Luomai28	15.7	CC	AA	CC	CC
40	Luomai44	13.9	TT	AA	TT	TT
41	Luomai45	14.2	CC	AA	TT	TT
42	Luomai50	14.7	CC	AA	CC	CC
43	Luomai56	14.8	TT	GG	CC	CC
44	Luoqing1905	15.4	TT	GG	TT	TT
45	Luoqingfeng2101	14.0	TT	GG	TT	TT
46	Luoqingmai109	13.5	TT	AA	TT	TT
47	Luoqingmai40	15.9	CC	GG	CC	CC
48	Luoqingmai69	14.0	TT	GG	CC	CC
49	Luoqingmai70	14.8	TT	GG	CC	CC
50	Nongxin699	15.7	TT	AA	TT	TT
51	Pingan18	15.3	TT	GG	CC	CC
52	Puling300	13.9	TT	GG	TT	TT
53	Pumai127	13.6	TT	GG	CC	CC
54	Pumai186	15.0	TT	GG	TT	TT
55	Puxing26	15.5	TT	AA	CC	TT
56	Puxing35	15.3	TT	AA	TT	CC
57	Ruihuamai513	16.6	TT	GG	TT	CC
58	Shangmai187	13.5	TT	GG	TT	TT
59	Shangmai198	13.2	CC	GG	CC	TT
60	Shanhe1029	15.0	CC	AA	CC	CC
61	Shi15-6375	13.9	TT	GG	TT	TT
62	Shunmai10	15.2	CC	AA	CC	CC
63	Taiheimai10	14.9	TT	GG	TT	TT
64	Tianmai116	15.6	CC	GG	TT	CC
65	Tianmai159	14.8	CC	AA	TT	CC
66	Tianmai196	14.1	TT	GG	TT	TT
67	Tianmin360	16.4	CC	AA	CC	CC
68	Tuomai303	13.6	TT	GG	TT	CC
69	Tuomai606	14.5	TT	GG	TT	TT
70	Wanfeng505	13.8	TT	GG	TT	CC
71	Wanke2mai1720	14.2	CC	GG	TT	TT
72	Wanke800	13.8	CC	GG	TT	TT
73	Wansu1617	14.6	TT	GG	CC	TT
74	Xianmai283	12.9	CC	GG	TT	TT
75	Xinkemai176	15.0	CC	GG	CC	CC
76	Xinkemai186	16.0	CC	GG	TT	TT
77	Xinmai26	14.9	TT	AA	CC	CC
78	Xinmai52	13.9	TT	GG	TT	TT
79	Xinmai65	14.8	CC	AA	CC	TT
80	Xinmai67	14.4	CC	GG	TT	CC
81	Xinmai72	14.5	CC	AA	TT	CC
82	Xinong1125	16.0	CC	AA	CC	CC
83	Xinong158	15.0	CC	AA	TT	TT
84	Xinong1871	14.4	TT	GG	TT	TT
85	Xinong20	16.0	CC	AA	CC	CC
86	Xinong5195	16.2	CC	AA	TT	TT
87	Xinong579	16.0	CC	GG	CC	CC
88	Xinong633	14.7	CC	AA	TT	TT
89	Xinong805a	16.0	CC	GG	CC	CC
90	Xinong857	14.7	CC	AA	TT	TT
91	Xinong867	14.3	CC	GG	TT	CC
92	Xinong926	13.5	TT	GG	CC	TT
93	Xinong9799	16.0	CC	AA	CC	CC
94	Xinshiji2	13.6	CC	GG	TT	TT
95	Xuke108	15.1	CC	AA	CC	CC
96	Xumai17106	13.7	CC	GG	TT	TT
97	Xumai2198	16.2	CC	GG	TT	CC
98	Yanbaol369	15.9	CC	AA	TT	CC
99	Yingqiang1	16.5	CC	AA	CC	TT
100	Yunong612	12.7	TT	GG	CC	CC
101	Yunong7928	14.4	TT	GG	TT	TT
102	Yunong907	14.8	CC	AA	TT	CC
103	Yunong912	14.9	CC	AA	CC	TT
104	Yunong917	15.6	CC	GG	CC	CC
105	Yuzhou121	15.3	CC	GG	CC	CC
106	Zhengda201	13.9	CC	AA	TT	CC
107	Zhengmai113	16.0	CC	AA	CC	CC
108	Zhengmai139	15.3	CC	AA	CC	TT
109	Zhengmai163	14.2	TT	GG	TT	TT
110	Zhengmai1926	14.6	CC	AA	CC	CC
111	Zhengmai2018	15.2	CC	AA	CC	CC
112	Zhengmai21	13.8	TT	GG	TT	TT
113	Zhengmai22	15.9	CC	AA	CC	TT
114	Zhengmai23	14.0	CC	GG	TT	TT
115	Zhengmai366	15.2	CC	AA	CC	CC
116	Zhengmai518	14.3	TT	AA	CC	TT
117	Zhengmai825	14.3	TT	GG	TT	CC
118	Zhengmai828	15.2	CC	AA	TT	TT
119	Zhengmai918	15.3	CC	AA	CC	TT
120	Zhengmai9699	17.1	CC	GG	CC	CC
121	Zhengyanmai182	13.8	TT	AA	TT	TT
122	Zhongmai255	16.2	CC	AA	TT	CC
123	Zhongnong031	15.1	CC	AA	CC	CC
124	Zhongnong539	13.4	TT	GG	TT	TT
125	Zhongyu1211	14.3	TT	AA	CC	TT
126	Zhongyu130	16.1	CC	GG	TT	CC
127	Zhongyu149	14.9	TT	GG	CC	CC
128	Zhongyu978	17.3	CC	AA	CC	CC
129	Zhongyuan26	16.7	CC	AA	CC	CC
130	Zhongzhimai13	14.4	CC	AA	TT	TT
131	Zhongzhu16	17.0	CC	GG	TT	CC
132	Zhoumai22	14.9	CC	AA	TT	TT
133	Zhoumai36	14.9	CC	GG	TT	CC
134	Zhumai566	13.9	TT	AA	TT	TT
135	Zhumai568	16.1	CC	AA	CC	CC
136	Zhumai569	14.4	CC	AA	TT	TT
137	Zhumai591	13.8	TT	GG	TT	TT
138	Zhumai592	15.4	CC	AA	CC	CC
139	Zhumai599	14.0	TT	AA	TT	CC
140	Zhumai762	14.6	TT	AA	CC	CC

Note: GPC values in this table represent cross-location and cross-year BLUE values of each wheat cultivar.
